# Cannabinoids as anticancer drugs: current status of preclinical research

**DOI:** 10.1038/s41416-022-01727-4

**Published:** 2022-03-11

**Authors:** Burkhard Hinz, Robert Ramer

**Affiliations:** Institute of Pharmacology and Toxicology, Rostock University Medical Centre, Schillingallee 70, 18057 Rostock, Germany

**Keywords:** Cancer, Drug discovery

## Abstract

Drugs that target the endocannabinoid system are of interest as pharmacological options to combat cancer and to improve the life quality of cancer patients. From this perspective, cannabinoid compounds have been successfully tested as a systemic therapeutic option in a number of preclinical models over the past decades. As a result of these efforts, a large body of data suggests that the anticancer effects of cannabinoids are exerted at multiple levels of tumour progression via different signal transduction mechanisms. Accordingly, there is considerable evidence for cannabinoid-mediated inhibition of tumour cell proliferation, tumour invasion and metastasis, angiogenesis and chemoresistance, as well as induction of apoptosis and autophagy. Further studies showed that cannabinoids could be potential combination partners for established chemotherapeutic agents or other therapeutic interventions in cancer treatment. Research in recent years has yielded several compounds that exert promising effects on tumour cells and tissues in addition to the psychoactive Δ^9^-tetrahydrocannabinol, such as the non-psychoactive phytocannabinoid cannabidiol and inhibitors of endocannabinoid degradation. This review provides an up-to-date overview of the potential of cannabinoids as inhibitors of tumour growth and spread as demonstrated in preclinical studies.

## Background

### History

The use of the cannabis plant for medicinal and ritual purposes dates back several thousand years. Accordingly, the psychoactive effect of cannabis is already mentioned in the Pen-ts’ao ching, the oldest pharmacopoeia in the world [1, 2]. Further evidence for a millennia-old use is based on cannabis-containing grave goods found in archaeological investigations of an Ukok ‘princess’ from the Pazyryk culture [3] or the remains of cannabis fruits identified in archaeobotanical investigations at the Laoguanshan cemetery from the Han dynasty in Chengdu, South China [4]. The introduction of cannabis into European medicine can be attributed to the Irish physician William B. O’Shaughnessy, who published a groundbreaking study on hemp in 1839 with his work ‘On the preparations of the Indian hemp or gunjah’ [5]. Although the isolation of a substance called ‘cannabinol’ from the exuded resin of Indian hemp dates back to a communication published in 1899 [6], it was not until the 1960s that Raphael Mechoulam and his collaborators published a series of studies that elucidated the chemical structure and activity of Δ^9^-tetrahydrocannabinol (THC), cannabidiol (CBD), and other cannabinoids [7–10].

### Endocannabinoid system

The entry of the endocannabinoid (EC) system into modern research as a potential target of pharmacotherapeutic intervention began with the discovery and cloning of specific G_i/o_ protein-coupled cannabinoid receptors, termed CB_1_ and CB_2_ [11, 12]. While CB_1_ receptors are primarily localised in the central nervous system, CB_2_ receptors are mostly expressed on cells of the immune system. Other components of the EC system discovered in the 1990s are N-arachidonoylethanolamine (anandamide, AEA) and 2-arachidonoylglycerol (2-AG), two endogenously synthesised agonists at cannabinoid receptors [13, 14]. Scientific attention has also been paid in recent years to substances with structural similarities to the aforementioned ECs, such as the cannabinoid receptor ligands 2-arachidonoyl glycerol ether (noladin ether), O-arachidonoylethanolamine (virodhamine), N-arachidonoyldopamine and oleic acid amide (oleamide) (reviewed in ref. [15]). However, the available data on their biological role are very limited. Furthermore, a number of N-acylethanolamines structurally similar to ECs, so-called EC-like substances, such as N-palmitoylethanolamine (PEA) and N-oleylethanolamine (OEA), have been described, which use the biosynthesis and degradation enzymes of ECs, but do not trigger cannabinoid receptor activation (reviewed in ref. [15]).

Around the turn of the millennium, the non-selective cation channel transient receptor potential vanilloid 1 (TRPV1) was described as an additional receptor target for several cannabinoids such as AEA [16] and the non-psychoactive phytocannabinoid CBD [17]. Among the phytocannabinoids, THC exhibits the properties of an agonist at the CB_2_ receptor and a partial agonist at the CB_1_ receptor [18], as well as an agonist at the G protein-coupled receptor (GPR) 55 [19]. In contrast to the high-affinity CB_1_ and CB_2_ receptor binding of THC with K_i_ values in the low nanomolar range, CBD has been demonstrated to have weaker affinities with K_i_ values in the micromolar range and non-competitive antagonistic effects at both CB_1_ and CB_2_ receptor [18]. In addition, CBD shows a binding preference to the CB_2_ receptor [20] and an antagonistic effect at GPR55 [19]. Among a variety of receptor interactions of CBD, the compound has been shown to increase the transcriptional activity of peroxisome proliferator-activated receptor γ (PPARγ) [21].

Other important elements affecting the tone of the EC system are EC-synthesising and -degrading enzymes. In this context, N-acyl-phosphatidylethanolamine-specific phospholipase D (NAPE-PLD), α/β-hydrolase domain-containing 4 (ABHD4), glycerophosphodiesterase-1 (GDE1), and tyrosine protein phosphatase non-receptor type 22 (PTPN22) have been described to contribute to AEA biosynthesis. Diacylglycerol lipase α and -β (DAGLα and -β) have been identified as 2-AG-producing enzymes (reviewed in ref. [22]). The degradation of AEA and 2-AG is endogenously mediated by the enzyme fatty acid amide hydrolase (FAAH) [23], whereas the hydrolysis of 2-AG proceeds mainly via monoacylglycerol lipase (MAGL) [24] with contribution of several other hydrolytic enzymes (ABHD6, ABHD12, FAAH). In addition, ECs can be degraded by enzymes of the arachidonic acid cascade, i.e. cyclooxygenase-2 (COX-2) and lipoxygenases (reviewed in ref. [22]).

It is worth noting that the definition of the EC system has been subject to evolution over time. Thus, the classical EC system only includes the two ECs AEA and 2-AG, their anabolic and catabolic enzymes, and the two cannabinoid receptors CB_1_ and CB_2_. However, as more became known about its complex networks over time, the term and concept of the EC system was expanded accordingly, leading to the definition of an “endocannabinoidome” [25]. This now includes also other EC-like lipid mediators, metabolic enzymes and novel cannabinoid targets such as GPRs (GPR18, GPR55, GPR119) and members of the transient receptor potential cation channel subfamily (TRPV1, TRPV2, TRPV4) (reviewed in ref. [15, 25]).

### Anticancer effect of cannabinoids—pioneering work and research strategies

The discovery of the anticarcinogenic effect of cannabinoid compounds can be dated back to Munson et al. [26], who were able to show in the mid-1970s that THC, Δ^8^-THC and cannabinol inhibit Lewis lung adenocarcinoma growth in mice. However, the discovery of cannabinoid receptors years later was only the starting point for an extensive and detailed investigation of the anticarcinogenic mechanisms of cannabinoid action. For example, a study at the turn of the millennium showed that intratumoural administration of THC and the synthetic cannabinoid agonist WIN 55,212-2 induced a considerable regression of malignant gliomas in Wistar rats and in mice deficient in recombination activating gene 2 [27]. A growth inhibitory effect of cannabinoids against glioma cells was confirmed shortly thereafter for the selective CB_2_ agonist JWH-133 [28]. With the discovery of a possible role of the EC system as a factor in cancer progression, numerous studies have also addressed a possible link between the regulation of cannabinoid receptors, ECs, and EC-synthesising and -degrading enzymes on the one side and disease severity and survival on the other (reviewed in ref. [15]). However, no clear correlation between the regulation of these parameters in tumour tissue and disease severity could be found, so that the aforementioned factors therefore do not show suitability as reliable biomarkers.

In addition to the activation of cannabinoid receptors by exogenously applied agonists, newer approaches to pharmacotherapeutic intervention by blocking EC turnover have been pursued and further developed in recent years, such as the blockade of MAGL by the selective MAGL inhibitor JZL184 [29]. In the context of potential cancer-preventive effects, knockdown or pharmacological inhibition of MAGL have been associated with decreased tumour cell invasion [30–32], metastasis [32] and tumour growth [30, 31]. This inhibitory effect on cancer progression by MAGL inhibition appears to be due to a dual-mode effect in prostate cancer cells involving both activation of CB_1_ receptor by increasing 2-AG levels and reduction of protumorigenic free fatty acids resulting from MAGL activity [30], whereas for the anti-invasive effect on lung cancer cells, only CB_1_ receptor involvement has been demonstrated [32]. On the other hand, a recent publication concluded that inhibition of MAGL promotes rather than retards cancer progression in mice and that knockout of MAGL is associated with increased incidence of lung adenocarcinoma [33]. With regard to the tumour suppressor function of MAGL, it was shown in this context that MAGL can inhibit the transactivation of epidermal growth factor receptor (EGFR)-associated signalling pathways as well as proinflammatory proteins such as COX-2 and tumour necrosis factor (TNF)-α. Accordingly, it is reasonable to assume that MAGL acts as a tumour suppressor or oncoprotein depending on the tissue type. Finally, inhibition of FAAH has also been shown to mediate cancer-limiting effects with growth inhibitory [34, 35] as well as anti-invasive and antimetastatic properties [36]. The preclinical work on the inhibition of FAAH and MAGL in cancer has been summarised elsewhere (reviewed in ref. [15]).

Recently, further substances from *Cannabis sativa* L., which are also found in other plants, have been investigated for their anti-cancer effects. This concerns, for example, the sesquiterpene β-caryophyllene, a potent CB_2_ agonist [37]. In the hitherto performed studies, β-caryophyllene showed antiproliferative and proapoptotic properties on various cancer cell lines [38, 39] and enhanced the cytostatic effects of classical chemotherapeutic agents such as doxorubicin [40] and sorafenib [41].

The first pilot clinical trial of cannabinoids as cancer treatment in 9 glioblastoma patients was published in 2006 and found that intracranially administered THC was safe [42]. Recently, the results of a randomised, placebo-controlled phase 1b study with nabiximols oromucosal spray (standardised extract of *Cannabis sativa* L. with an approximate 1:1 ratio of THC and CBD) in combination with dose-intense temozolomide in patients with recurrent glioblastoma multiforme were published [43]. Of the 21 patients in this study, survival at one year was 83% in the 12 patients treated with nabiximols, while it was significantly lower at 44% in the 9 patients treated with placebo, leading the authors to recommend further exploration in an adequately powered randomised controlled trial. So far, however, randomised, placebo-controlled studies with a larger number of cancer patients are lacking at all.

**Fig. 1:**
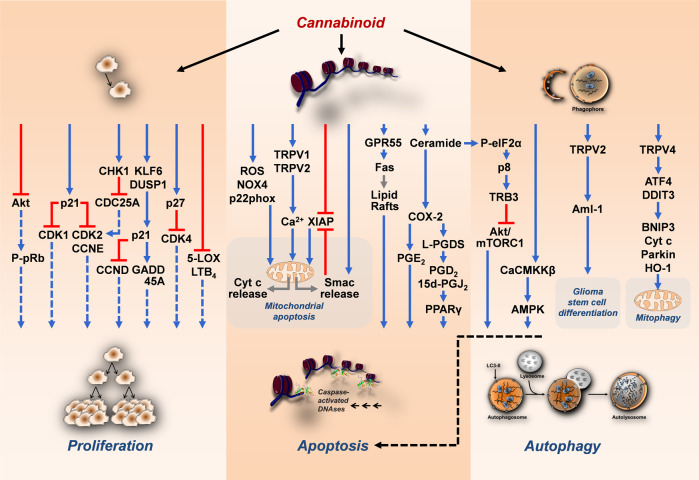
Mechanisms of antiproliferative, proapoptotic and proautophagic effects of cannabinoids on cancer cells. The black arrows emanating from the cannabinoid show the respective modulated structures or levels. Coloured arrows indicate inhibitory (red) and stimulatory (blue) effects of cannabinoids on the indicated targets. Blue dashed arrows indicate reduced stimulation of the respective effect by cannabinoid treatment. The grey arrows indicate a shift in a parameter. The black dashed arrow indicates a functional relationship between autophagy and apoptosis. All abbreviations are explained in the text.

**Fig. 2:**
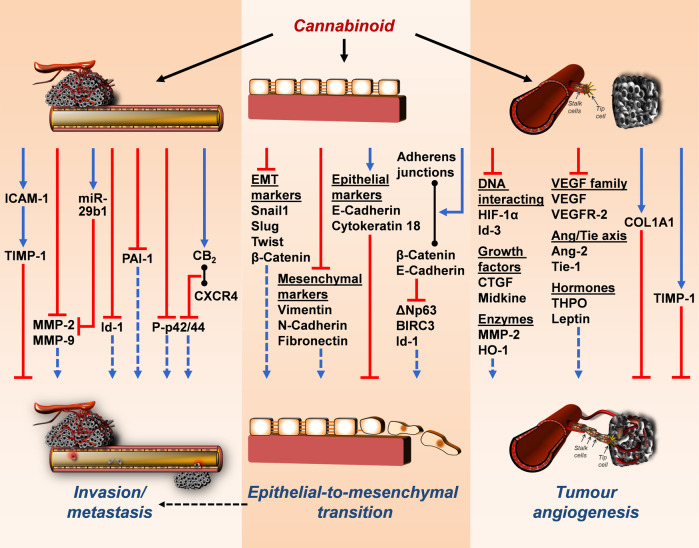
Mechanisms of anti-invasive, antimetastatic, anti-epithelial-to-mesenchymal-transition and anti-angiogenic effects of cannabinoids on cancer cells. The black arrows emanating from the cannabinoid show the respective modulated structures or levels. Coloured arrows indicate the inhibitory (red) and stimulatory (blue) effects of cannabinoids on targets involved in cancer cell invasion/metastasis, angiogenesis and epithelial-to-mesenchymal transition. Blue dashed arrows indicate reduced stimulation of each effect by cannabinoid treatment. Black lines with circles at both ends indicate binding and dimer formation between the respective parameters. Black dashed arrow indicates functional relationship between epithelial-to-mesenchymal transition and invasion/metastasis. All abbreviations are explained in the text.

The following chapters provide an overview of the preclinical evidence on the effect of cannabinoids on cancer growth and metastasis, with the most relevant cannabinoid targets summarised in Fig. 1 (tumour cell proliferation, apoptosis and autophagy) and Fig. 2 (tumour cell invasion, angiogenesis).

## Tumour cell proliferation, apoptosis and autophagy

Publications on growth inhibitory effects of cannabinoids have accumulated over the last two decades. The dominant cellular model used for this purpose in the 2000s was glioma cells. However, over time, a wide range of different tumour cell lines of various entities have been tested. Whereas early work focused on mechanisms leading to cannabinoid-induced apoptosis and cell cycle arrest of cancer cells, these investigations were complemented later by studies dealing with autophagy effects. Due to the large amount of data, only a selection could be made here.

### Tumour cell proliferation

As mentioned earlier, the description of the tumour regressive effects of THC and WIN 55,212-2 on rat and mouse glioma xenografts represents the first comprehensive study in this century investigating cannabinoids as potential anticancer drugs [27]. Since then, several mechanisms by which cannabinoids inhibit tumour growth have been elucidated. Among these, inhibition of protein kinase B (Akt) appears to be an important mechanism. Accordingly, the antiproliferative action of cannabinoids on melanoma cells has been linked to cell cycle arrest at the G_1_/S transition via inhibition of the prosurvival protein Akt and hypophosphorylation of the retinoblastoma protein (pRb) tumour suppressor protein [44]. Furthermore, CB_2_ receptor-mediated inhibition of Akt has been reported in ErbB2-positive breast cancer progression in mouse mammary tumour virus (MMTV)/neu transgenic mice as a syngeneic tumour model [45]. Meanwhile, publications have confirmed that inactivation of the Akt pathway is also involved in the antitumour activity of cannabinoids on human gastric cancer [46], non-small cell lung cancer [47] and hepatocellular carcinoma (HCC) cells [48]. In breast cancer cells, features of cell cycle arrest induced by cannabinoid treatment include blockade of the G_1_/S transition through downregulation of cyclin-dependent kinase 1 (CDK1), induction of p21 [49], or induction of p27kip1, decrease in cyclin (CCN) A and E, degradation of cell division cycle 25 A (CDC25A) and inactivation of CDK2 [50]. Furthermore, in prostate cancer cells, WIN 55,212-2 was shown to reduce proliferation and to arrest cells in the G_0_/G_1_ phase via CB_2_ receptor-dependent signalling [51]. Studies of the underlying mechanism showed an upregulation of p27 and a reduction in CDK4 expression and phosphorylated pRb (P-pRb) when cells were treated with the cannabinoid.

CBD appears to inhibit cancer cell proliferation primarily via apoptosis signalling. Thus, in a recent study [52], CBD suppressed the proliferation and growth of head and neck squamous cell carcinomas by inducing the apoptotic and autophagy activity of DUSP1 (Dual Specificity Phosphatase 1), which is known to interfere as a negative regulator with EGFR-initiated mitogen-activated protein kinase (MAPK) signalling and associated proliferation. Furthermore, increased expression levels of p21, Kruppel-like factor 6 (KFL6) and growth arrest and DNA damage-inducible protein α (GADD45A) were observed, for which antiproliferative effects were also described. Another recent study investigating the in vitro effects of CBD on human gastric cancer cells revealed an antiproliferative effect accompanied by significant upregulation of ataxia telangiectasia mutated (ATM) gene and p21 protein expression and downregulation of p53 protein expression, which subsequently resulted in decreased levels of CDK2 and CCNE and cell cycle arrest in the G_0_/G_1_ phase [53]. Finally, a contribution of the eicosanoid system was also supported by the observation that CBD reduced the activity and content of 5-lipoxygenase (5-LOX) and its end product leukotriene B_4_ in the tumour tissue of nude mice xenografted with human glioma cells [54]. In addition, a synergism of the viability-reducing effect of CBD and the 5-LOX inhibitor MK-886 could be shown in vitro, which also argues for a modulatory effect of 5-LOX on glioma cell fate.

Conversely, a recent study has demonstrated that the CB_1_ agonist arachidonyl-2’-chloroethylamide (ACEA), the selective CB_2_ agonist HU308 and THC promoted rather than inhibited the progression of human papillomavirus-positive squamous cell carcinomas of the head and neck, with THC-treated tumour xenografts growing faster than controls [55]. This work is in line with previous in vitro studies by other authors [56–58], who also found proliferative effects on cancer cells triggered by submicromolar concentrations of various cannabinoids, with corresponding THC effects associated with transactivation of EGFR in one paper [56]. Some other studies, in contrast, failed to observe mitogenic effects of THC when nanomolar concentrations were tested [42, 59, 60]. Overall, however, these results seem worth reconsidering, as peak plasma concentrations of THC after inhalation or oral administration do not indeed exceed 1 μM [55]. In fact, many experimental studies investigating proapoptotic effects of cannabinoids have been conducted with concentrations above 1 µM, which, on the other hand, could well be achieved with intratumoral administration [27]. Interestingly, hardly any mitogenic effects can be observed in the case of CBD (reviewed in ref. [61]).

### Tumour cell apoptosis

An important role in cannabinoid-induced apoptosis is played by the proapoptotic sphingolipid ceramide. Initial studies showed that THC and other cannabinoids cause the death of glioma cells via cannabinoid receptor-dependent de novo synthesis of ceramide [27, 28, 62]. The importance of ceramide for cannabinoid-induced cytotoxic autophagy was discovered later in 2009 [63]. Further apoptosis mechanisms were described with CB_2_ receptor-dependent induced apoptosis of glioma cells associated with an increase in the stress-associated transcriptional coactivator p8 as an upstream regulator of the endoplasmic reticulum (ER) stress-related proteins activating transcription factor (ATF)-4 and tribbles pseudokinase 3 (TRB3) [64]. A similar apoptotic effect was found for CB_1_ receptor-induced growth inhibition in response to Akt inhibition in translocation-positive rhabdomyosarcoma cells [65]. Here, cannabinoid-induced loss of viability was suppressed by transfection of p8 siRNA.

Depending on the substance and cell type, the eicosanoid system also revealed to play an important role in the proapoptotic effect of cannabinoids. Thus, in temporal relation to the first mechanistic findings on cannabinoid-induced glioma cell death, ceramide synthesised de novo by *R*(+)-methanandamide (Met-AEA) in neuroglioma cells was shown to lead to an induction of COX-2 expression [66], which contributes to Met-AEA-induced cell death via proapoptotic prostaglandin (PG) E_2_ [67]. Thereby, Met-AEA-triggered apoptosis was found to be independent of cannabinoid receptor and TRPV1 activation. The influence of COX-2 on cancer cell fate was also addressed in follow-up work on the effect of Met-AEA [68] and the established chemotherapeutic agents paclitaxel, cisplatin and 5-fluorouracil [69] on human cervical carcinoma cells. Here, the PPARγ-activating eicosanoids PGD_2_ and 15-deoxy-Δ^12,14^-PGJ_2_ could be identified as apoptosis mediators, whereby post-transcriptional knockdown of COX-2, downstream lipocalin-type PGD synthase (L-PGDS) and transcription factor PPARγ led to inhibition of apoptosis triggered by Met-AEA and chemotherapeutic agents. A functional role of the aforementioned PPARγ-activating PGs could also be proven for the CBD-triggered cannabinoid receptor and TRPV1-independent apoptotic death of lung cancer cells [70]. CBD also led to an upregulation of COX-2 and PPARγ in tumour tissue in A549-xenografted nude mice and to tumour regression, which was reversed by a PPARγ antagonist [70].

As already mentioned above, cannabinoid receptors CB_1_ and CB_2_ do not necessarily have to be involved in the apoptosis induction of a cannabinoid. In a previous study, AEA was demonstrated to induce apoptotic death of human neuroblastoma and lymphoma cells via TRPV1, which was paralleled by an increase in intracellular calcium, mitochondrial uncoupling and cytochrome c (Cyt c) release [71]. In other studies, AEA has been shown to induce receptor-independent apoptosis of non-melanoma cancer cells via ER stress [72] and to elicit reduced viability of cholangiocarcinoma cells via activation of GPR55, with the latter eliciting increased recruitment of the death receptor Fas in membrane lipid rafts [73]. Furthermore, activation of TRPV2 has been revealed to be involved in the proapoptotic effect of CBD on human bladder cancer cells [74]. Mostly, however, the proapoptotic effects of CBD have been associated with receptor-independent mechanisms, although corresponding inhibitor experiments with receptor antagonists have not always been performed. This applies, for example, to some studies in which CBD elicited apoptosis of mammary carcinoma [75] and human gastric cancer cells [53] via the formation of reactive oxygen species (ROS). Interestingly, however, CBD induced a CB_2_ receptor-dependent mitochondrial apoptosis in human leukaemia cells, which was accompanied by ROS production, increased expression of the NAD(P)H oxidases Nox4 and p22phox and release of Cyt c [76]. In a recently published study [77], CBD was also shown to promote apoptosis of gastric cancer cells by suppressing X-linked inhibitor apoptosis (XIAP), a member of the IAP protein family. Thereby, CBD reduced XIAP protein levels while increasing ubiquitination of the protein. In addition, CBD increased the interaction between XIAP and Smac by inducing the release of Smac from mitochondria into the cytosol and promoted mitochondrial dysfunction. Finally, recent evidence suggests that CBD at a relatively high concentration of 30 µM switches the mitochondrial voltage-dependent anion channel (VDAC) from fully open to major subconductance state, thereby arresting the Ca^2+^-permeable state of this channel and inducing a severe oxidative stress, mitochondrial Ca^2+^ overload, Cyt c release into the cytosol as well as induction of LC3-phosphatidylethanolamine conjugate (LC3-II) and caspase activation in leukaemia cells [78].

### Tumour cell autophagy

Autophagy can lead to survival or death of cells. Research in recent years has shown that autophagy signalling pathways may also play an important role in the toxicity of cannabinoid compounds on cancer cells. Initial experiments conducted by Salazar et al. [63] demonstrated that THC induces death of human glioma cells via stimulation of autophagy. In this process, THC led to ceramide accumulation and phosphorylation of eukaryotic translation initiation factor 2α (eIF2α), a subsequent ER stress response and, via this, finally to the induction of autophagy via the TRB3-dependent inhibition of the Akt/Mammalian Target of Rapamycin Complex 1 (mTORC1) axis. In the processes described, autophagy preceded apoptosis. In another work, proautophagic effects of THC and the CB_2_ agonist JWH-015 in human HCC cells were reported [79]. Here, autophagy was due to upregulation of TRB3 and subsequent inhibition of the Akt/mTORC1 axis and adenosine monophosphate-activated kinase (AMPK) stimulation, with Ca^2+^/calmodulin-activated kinase kinase β (CaCMKKβ) being responsible for cannabinoid-induced AMPK activation and autophagy. Importantly, the tumour regressive effects of THC and JWH-015 on subcutaneous HCC xenografts were abolished in vivo when autophagy was genetically or pharmacologically inhibited [79]. A functional link between autophagic signalling pathways and cannabinoid-induced apoptosis was confirmed by several other studies. Accordingly, in melanoma cells, co-treatment of THC with the autophagic flux inhibitor chloroquine or knockdown of autophagy-related 7 (Atg7), an essential molecule for induction of autophagy, resulted in suppression of THC-induced autophagy as well as cell death [80]. Consistent with this, inhibition of CBD-induced autophagy by chloroquine also led to a significant increase in the viability of CBD-treated human squamous cell carcinomas of the head and neck [52].

On the other side and in contrast to previous studies on glioma cells, a recent study reported that knockdown of autophagy genes led to enhancement of WIN 55,212-2-induced apoptotic cell death of human glioblastoma cells [81]. The authors concluded that autophagy induced by cannabinoid treatment is a protective mechanism and autophagy inhibitors may be potential agents to enhance cannabinoid action. Evidence of cannabinoid-protective autophagy was also provided by a glioblastoma cell culture study in which the addition of chloroquine led to an increase in CBD-induced cell death [82].

In glioma stem cells, a cell subpopulation of glioblastoma multiforme implicated in chemoresistance, CBD was further shown to induce a TRPV2-dependent autophagic process that stimulates glioma stem cell differentiation via induction of a splice variant of the acute myeloid leukaemia transcription factor (Aml-1), thereby abrogating their chemoresistance to carmustine [83]. In breast cancer cells, CBD-induced intrinsic apoptosis was associated with autophagy and decreased levels of mTOR, its downstream effector eukaryotic initiation factor 4E binding protein 1 (4EBP1) and CCND1 [75]. Finally, in a recent study, a significant increase in LC3-II levels as an autophagy marker was observed in two out of three medulloblastoma cell lines after CBD and THC exposure, which was accompanied by an increase in poly(ADP-ribose) polymerase (PARP) cleavage [84]. However, these findings did not translate to in vivo models in mice, as the survival rate of the animals did not change.

As another cannabinoid-triggered upstream ion channel a recent investigation found TRPV4 and a signalling pathway including ATF4, DNA Damage Inducible Transcript 3 (DDIT3), TRB3, Akt and mTOR as mediators of CBD-induced mitophagy in glioma cells [85]. Mitophagy involves the selective degradation of mitochondria by autophagy and is manifested in mitophagy-related proteins such as BCL2 interacting protein 3 (BNIP3), Cyt c, Parkin and engulfed mitochondria, with mitochondrial dysfunction and overactivation of heme oxygenase-1 (HO-1) acting synergistically in lethal mitophagy [86].

## Tumour cell invasion and metastasis

### Tumour cell invasion

A large number of publications also point to an inhibitory effect of cannabinoid compounds on tumour cell migration, invasion and in vivo metastasis (Fig. 2). The first report on this referred to the CB_1_ receptor-dependent inhibition of prostate cancer cell invasion by the EC 2-AG [87]. Anti-invasive actions were also later proven for AEA in glioma [88] and lung cancer cells [36]. A few years earlier, the AEA derivative Met-AEA had already been shown to exhibit anti-invasive properties on human cervical and lung cancer cells via cannabinoid receptors and TRPV1 [89].

In one of the first mechanistic studies in this field, induction of tissue inhibitor of metalloproteinase-1 (TIMP-1) expression was shown to be a cause of the anti-invasive effect of THC, Met-AEA and CBD in cervical and lung cancer cells, with cannabinoid receptors (THC, Met-AEA, CBD) and TRPV1 (Met-AEA, CBD) mediating this response [89, 90]. Crucially, TIMP-1 inhibits collagen-degrading enzymes such as matrix metalloproteinase (MMP)-2 and MMP-9, which play an important role in promoting cancer metastasis (reviewed in ref. [91]). Accordingly, overexpression of TIMP-1 has been associated with a reduction in tumour growth, cancer cell invasiveness and metastasis (reviewed in ref. [92]). An anti-invasive effect based on TIMP-1 induction in lung cancer cells was also confirmed for the FAAH inhibitors N-arachidonoyl-serotonin (AA-5HT) and URB597, AEA and OEA [36] as well as for the MAGL inhibitor JZL184 and the MAGL substrate 2-AG [32]. For the anti-invasive effect of THC, CBD and Met-AEA on lung tumour cells, a cannabinoid-mediated increased expression of the intercellular adhesion molecule-1 (ICAM-1) as an upstream inducer of TIMP-1 expression could be demonstrated [93].

In support of the importance of modulation of extracellular matrix proteolysis in anti-invasive cannabinoid action, other groups have demonstrated downregulation of MMP-2 by THC in glioma cells [94] and downregulation of MMP-2 and -9 in HCC cells treated with CB_1_ (ACEA) or CB_2_ agonists (CB65) [95]. Notably, the antimigratory effect of the synthetic cannabinoid WIN 55,212-2 on osteosarcoma cells was associated with downregulation of MMP-2 and -9 and a 700-fold upregulation of miR-29b1, a key miRNA that downregulates MMP-2 and -9 [96]. A recent study also focused on the role of cancer-associated fibroblasts as a crucial element of the stromal compartments in the tumour microenvironment. Here, conditioned media from WIN 55,212-2-treated cancer-associated fibroblasts were shown to impair the invasive properties of prostate cancer cells due to a cannabinoid-mediated reduction in MMP-2 release [97].

In breast cancer, glioblastoma and salivary gland cancer cells, a CBD-mediated downregulation of Id-1, an inhibitor of basic helix-loop-helix transcription factors, has been reported as the cause of the anti-invasive effect of this compound [98–100]. CBD also led to a downregulation of the sex-determining region Y (SRY)-Box 2 (Sox-2), a critical determinant of glioma tumour initiating cell growth and downstream target of Id-1, in glioblastoma cells [99]. Finally, the antimetastatic effect on breast cancer cells demonstrated for CBD in a mouse model [101] was directly linked to a downregulation of Id-1 in a later work [102]. In another investigation, siRNA, inhibitor and add-back experiments showed that the cannabinoid receptor- and TRPV1-dependent downregulation of plasminogen activator inhibitor (PAI)-1 in CBD-exposed lung cancer cells [103] is part of the anti-invasive effect of this cannabinoid, in addition to the upregulation of TIMP-1 mentioned above. Still other work revealed that the anti-invasive effect of THC on cholangiocarcinoma cells is associated with reduced activation of Akt and p42/44 MAPK [104]. Furthermore, a very recent work found that treatment with CBD in combination with THC or CBD alone inhibited bladder urothelial carcinoma cell migration independently of cannabinoid receptors [105].

Contradictory results have been published on the role of the CB_2_ receptor in the invasion process. While studies with selective CB_2_ agonists [95, 102] as well as inhibitor studies [36, 89, 90, 93, 103] suggest a role for CB_2_ in the anti-invasive effects of various cannabinoids, a recent investigation reported that silencing of the CB_2_ receptor reduced proliferation, migration and invasion of lung cancer cells, which was associated with reduced levels of phospho-Akt, phospho-mTOR and decreased expression of 70-kDa ribosomal protein S6 kinase (p70S6K), a mitogen-activated Ser/Thr protein kinase that promotes cell survival and growth [106]. With respect to the CB_2_ receptor, it has also been shown that the latter can form an induced heterodimer with the G protein-coupled chemokine receptor CXCR4 in human breast and prostate cancer cells [107]. Here, simultaneous agonist-dependent activation of CXCR4 and CB_2_ resulted in reduced CXCR4-mediated formation of phosphorylated p42/44 MAPK (P-p42/44) and ultimately diminished cancer cell chemotaxis. Therefore, cannabinoids could also negatively modulate tumour progression by interfering with CXCR4 receptor function.

A number of other publications addressed the role of epithelial-mesenchymal transition (EMT)-lowering properties of cannabinoid compounds as a contributing mechanism of action to reduce cancer aggressiveness (Fig. 2). An early study on this topic showed that the AEA derivative 2-methyl-2′-F-anandamide (Met-F-AEA) reduced cytoplasmic and nuclear protein levels of β-catenin, one of the key factors involved in the EMT transition [108]. In this publication, Met-F-AEA further caused significant inhibition of mesenchymal markers such as vimentin, N-cadherin, fibronectin and EMT markers (Snail1, Slug and Twist) and upregulation of epithelial markers such as E-cadherin and cytokeratin 18. Recently, CBD was also shown to reverse interleukin (IL)-1β-induced EMT of human breast cancer cells by reprogramming invasive cells into cells with a non-invasive phenotype [109]. Here, CBD induced the relocalisation of E-cadherin and β-catenin at adherens junctions, thereby preventing nuclear translocation of β-catenin and inhibited the expression of the EMT marker ΔNp63, an isoform of tumour protein 63 (TP63), baculoviral IAP repeat-containing protein 3 (BIRC3) and Id-1. In the same work, studying the malignant phenotype of breast cancer cells, CB_1_ receptor-mediated inhibition of viability by CBD was registered, as well as an antimigratory effect of the cannabinoid associated with inhibition of Akt phosphorylation [109]. Others observed a reversal of transforming growth factor (TGF)-β-induced spindle-shaped morphology of lung cancer cells corresponding to the reorganisation of the stress fibre F-actin when the cells were treated with a combination of THC and CBD [110]. In this study, TGF-β-induced inhibition of E-cadherin expression and upregulation of N-cadherin and vimentin were significantly reversed as a characteristic EMT regulatory pattern in the presence of CBD, THC or the combination of both. Finally, these cannabinoid-mediated regulations were functionally linked to a reduced migration potential of lung cancer cells.

### Metastasis

The effect of cannabinoids in experimental metastasis models has been described in detail elsewhere (reviewed in ref. [111]). In short, phytocannabinoids (THC, CBD), ECs and EC analogue (AEA, 2-AG, Met-F-AEA), EC-like substances (OEA, PEA) and EC degradation inhibitors (AA-5HT, URB597, JZL184) showed an inhibitory effect on metastatic infiltration of the lung with previously injected lung carcinoma cells [32, 36, 90, 93, 112, 113]. In addition, inhibitory effects of cannabinoids on breast cancer [101, 102, 114], salivary gland cancer [100] and melanoma cell metastasis [44] have been described. Finally, further work showed that JZL184 impairs bone metastasis of osteotropic prostate and breast cancer cells in mice and inhibits metastasis of osteosarcoma cells [115] and that knockdown of MAGL is associated with reduced lymph node metastasis in MAGL-overexpressing nasopharygeal carcinoma cells [116].

Regarding the underlying mechanisms, one investigation demonstrated increased ICAM-1 expression as a cause of the antimetastatic effect of CBD on lung cancer cells, with the effect being reversed by an ICAM-1 neutralising antibody [93]. Another work showed that CBD inhibited lung metastasis of breast cancer cells expressing a control vector but not an Id-1-containing vector [102]. Further studies have identified the CB_1_ receptor as an initial platform of the antimetastatic effect of Met-F-AEA [112] and JZL184 [32], as the inhibition of metastasis induced by these compounds was counteracted by CB_1_ receptor antagonists.

## Tumour angiogenesis

Several studies have shown that cannabinoid compounds inhibit tumour neovascularisation in mouse models with xenografts (reviewed in ref. [117]). However, data on the exact mechanisms underlying these effects, especially with regard to tumour-stroma interactions, are still scarce. Early reports on the effect of cannabinoid compounds on tumour formation found a regressive effect based on anti-angiogenic effects through the downregulation of a number of proangiogenic parameters such as vascular endothelial growth factor (VEGF), placental growth factor (PlGF), angiopoietin-2 (Ang-2) [118, 119] and MMP-2 [119]. The same group later revealed that the CB_2_ receptor agonist JWH-133 modulates several hypoxia-related angiogenesis markers, most of which are associated with the VEGF pathway [120]. Thus, downregulation of VEGF-A and -B, hypoxia-inducible factor-1α (HIF-1α), connective tissue growth factor (CTGF), midkine, Id-3, Ang-2 and its receptor tyrosine kinase with immunoglobulin-like and EGF-like domains 1 (Tie-1), and HO-1 was detected, while upregulation was found for the type I procollagen α1 chain (COL1A1) [120]. Remarkably, the authors of the latter study found VEGF receptor (VEGFR)-2 downregulation in response to JWH-133 in experimental glioma xenografts in mice. In another paper, inhibition of MMP-2 expression in human umbilical vein endothelial cells (HUVEC) was confirmed as part of the anti-angiogenic effects of CBD [121]. Consistent with the aforementioned direct anti-angiogenic effects, a further investigation identified the hexahydrocannabinol analogues LYR-7 [(9S)-3,6,6,9-tetramethyl-6a,7,8,9,10,10a-hexahydro-6H-benzo[c]chromen-1-ol] and LYR-8 [(1-((9S)-1-hydroxy-6,6,9-trimethyl-6a,7,8,9,10,10a-hexahydro-6H-benzo[c]chromen-2-yl)ethanone)] as inhibitors of VEGF-induced proliferation, migration and capillary-like tube formation of human endothelial cells as well as VEGF-induced blood vessel formation in the chorioallantoic membrane assay and VEGF release from cancer cells [122]. Thereby, the effects of the test substances on cell proliferation and tube formation were not abolished by cannabinoid receptor antagonists, suggesting a cannabinoid receptor-independent mechanism.

A number of studies have also demonstrated indirect effects of cannabinoids on endothelial cells through cannabinoid-mediated modulation of the secretome of tumour or other non-endothelial cells. Thus, conditioned media from AEA-treated breast cancer cells showed an inhibitory effect on endothelial cell proliferation associated with an approximate halving of the VEGF concentration, but also in a significant reduction of other proangiogenic factors such as leptin and thrombopoietin (THPO) [123]. A further indirect effect, here mediated by cannabinoid-induced increased formation of anti-angiogenic TIMP-1, was reported for the effect of conditioned media from CBD-, THC-, Met-AEA- and JWH-133-treated lung cancer cells on tube and sprout formation and endothelial cell migration [124]. A recent report also dealt with the effect of cannabinoids on human neutrophils with special regard to the regulation of angiogenic factors. Thereby, submicromolar concentrations of ACEA and JWH-133 inhibited lipopolysaccharide (LPS)-induced VEGF-A release, which was accompanied by a decrease in LPS-induced angiogenic effects on bovine aortic endothelial cell tube formation [125].

Divergent findings have been published regarding the direct effects of cannabinoid compounds on endothelial cells, which depend on the cellular system, the substance and the concentration used. Thus, in a previous study, inactivation of the CB_1_ receptor with the antagonist SR141716 as well as CB_1_ knockdown using siRNA caused inhibition of basic fibroblast growth factor (bFGF)-induced proliferation, migration and capillary-like tube formation of HUVEC [126] through pathways involving decreased activation of focal adhesion kinase, c-Jun N-terminal kinase, RhoA and MMP-2. Moreover, CB_1_ antagonism was confirmed in vivo to inhibit bFGF-induced neovascularisation in the rabbit cornea [126]. As for the direct effects of ECs on endothelial cells, it has been shown that bFGF-stimulated proliferation of HUVEC is upregulated by nanomolar concentrations of AEA [126], while in another work of the same group, micromolar proapoptotic concentrations of the AEA analogue Met-F-AEA inhibited bFGF-stimulated proliferation of porcine endothelial cells in a CB_1_ receptor-dependent manner [127]. In a further study, no anti-angiogenic effects of ECs on endothelial proliferation were observed and proangiogenic effects of THC and CBD were found at nanomolar concentrations [128]. Others reported that CBD, THC, Met-AEA and JWH-133 (3 µM each) exhibited pro- rather than anti-angiogenic effects in HUVEC directly exposed to these substances [124]. Finally, AEA was demonstrated to induce endothelial cell tube formation and proliferation, with both effects being reversed by inactivation of TRPV1 [129]. The authors assumed a TRPV1-dependent uptake of AEA into endothelial cells, which subsequently causes a proangiogenic effect on endothelial cells via the activation of intracellular cannabinoid receptors.

In terms of the direct influence of CBD on the endothelium, a recent study should also be mentioned which showed that CBD promotes ROS-dependent HO-1 expression in HUVEC, followed by HO-1-dependent cytoprotective autophagy [130]. This protection was maintained up to a certain CBD concentration (in the cited study up to 6 µM), but was then no longer sufficient to protect the cells from apoptotic cell death, which was also HO-1-dependent. The proapoptotic concentration of 10 µM CBD registered in this work is within the concentration range of the previously shown anti-angiogenic effects of CBD at concentrations ≥ 9 µM [121]. In the latter study, however, cytostasis, but not the induction of apoptosis, was associated with the decrease in metabolic activity. In this context, the use of different supplements leading to phenotypic heterogeneity [131] could explain the study-dependent varying sensitivity of HUVEC to high CBD concentrations.

## Interactions with the immune system

**Fig. 3:**
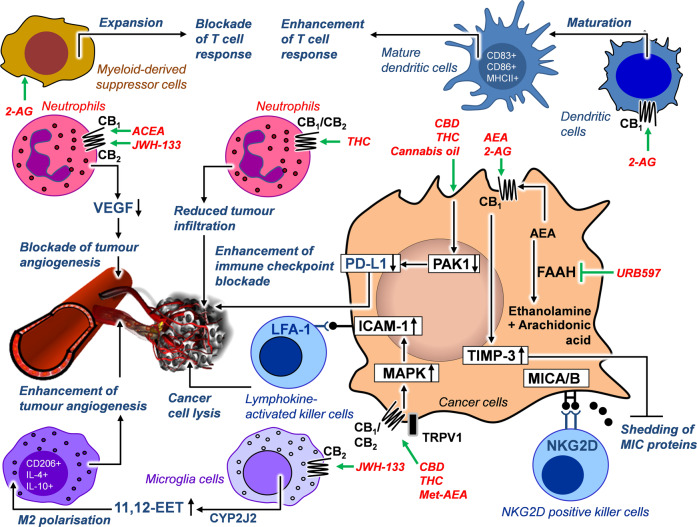
Effects of cannabinoid compounds on tumour-immune interaction. Green arrows indicate the specific site of action of the indicated cannabinoids. Black lines with circles at the end indicate receptor interactions. Black arrows indicate a functional or regulatory consequence of cannabinoid treatment. The indication “CB_1_/CB_2_” means that the substances listed here act via both cannabinoid receptors. All abbreviations are explained in the text.

As stromal cells in the tumour microenvironment are targets for therapeutic intervention, the role of immune cells as an important part of the tumour stroma has been brought into focus in recent years in relation to cannabinoid effects on cancer progression (Fig. 3). In this context, single-cell transcriptome profiling revealed that THC significantly affects transcriptomic subclusters in immune cell types [132]. A recent study showed inhibition of Kras-activated signalling pathways by p21 activated kinase 1 (PAK1) after treatment of cells with CBD and THC. Cannabinoids were found to reduce expression of programmed death ligand 1 (PD-L1) via decreasing PAK1 activity, thereby enhancing immune checkpoint blockade of pancreatic cancer cells [133]. For other peripheral cells, such as neutrophil granulocytes, their ability to produce proangiogenic factors such as VEGF after exposure to LPS was shown to be blocked by treatment with CB_1_ (ACEA) and CB_2_ agonists (JWH-133), which in turn led to decreased tumour growth via inhibition of angiogenesis [125].

Regarding the effect of ECs, one study investigated the influence of 2-AG on different subpopulations of immune cells involved in the progression of pancreatic ductal adenocarcinoma using an orthotopic mouse model. Here, 2-AG increased the proportion of CD83^+^, CD86^+^ and MHCII^+^ cells in CD11C^+^ cell populations in the spleen of mice [134]. Dendritic cells promoted to maturation by 2-AG exhibited higher expression of proinflammatory cytokines (IL-6, IL-12, interferon-α) mediated by activation of the CB_1_ receptor and subsequent upregulation of the phosphorylated form of signal transducer and activator of transcription 6 (STAT6). A concomitant activation of T cells in the spleen was not observed. On the other hand, in spleen and tumour tissue of mice, 2-AG also induced the proliferation of myeloid-derived suppressor cells, which are known to suppress the T cell response thereby promoting an immunosuppressive microenvironment. Nevertheless, the antiproliferative effect of 2-AG on cancer cells ultimately led to an overall tumour-regressive effect in vivo [134].

In vivo experiments with murine melanoma models indicated that WIN 55,212-2 inhibited cancer growth more efficiently in immunocompetent compared to immunodeficient mice [44]. A further in vivo study reported reduced infiltration of macrophages and neutrophils into experimental skin tumours after treatment of mice with THC [135]. As THC did not inhibit cancer cell proliferation in this work, the authors concluded that THC causes inhibition of cancer growth in vivo due to its cannabinoid receptor-activating properties in the tumour microenvironment and not in the tumour itself. In a further study the in vitro interaction of lung cancer cells with lymphokine-activated killer cells was addressed [136]. Here, prior treatment of cancer cells with CBD, THC and Met-AEA led to increased killer cell-mediated lysis of lung cancer cells. As a cause, an ICAM-1 upregulation on tumour cells induced by the tested cannabinoids could be identified with the consequence of an increased cross-linking with the lymphocyte function-associated antigen-1 (LFA-1) on the surface of natural killer cells [136]. Yet another work showed that inhibition of endogenous AEA degradation enhanced interaction with the tumour immune system. Accordingly, the FAAH inhibitor URB597 suppressed the shedding of the proteins major histocompatibility complex class I polypeptide-related sequence A (MICA) and B (MICB) on the surface of human HCC cells [137]. Since MICA/B proteins are recognised by cytotoxic lymphocytes expressing the natural killer group 2D (NKG2D) receptor and tumour cells are subsequently eliminated, preventing the shedding of MICA/B proteins can improve antitumour immunity. In the paper presented here, the effect was indirect, and was shown to be due to increased expression of TIMP-3. The authors further found AEA, 2-AG and the CB_2_ receptor agonist AM1241 to enhance TIMP-3 expression accompanied be a reduction of MICA/B shedding. Noteworthy, the TIMP-3 inducing and inhibitory action on MICA/B shedding upon treatment with URB597 was partially prevented by the CB_1_ receptor antagonist rimonabant.

In contrast to these positive effects, however, another early study on this topic reported that THC increases breast cancer growth and promotes tumour cell spread by inhibiting the antitumour immune response via enhancement of Th2-associated cytokines [138]. However, the study design used here was to exclude the direct growth inhibitory effect of cannabinoids on tumour cells in order to focus on the cannabinoid effect on the immune system. For this reason, tumour cells whose expression of cannabinoid receptors was low to undetectable were used in the experiments. This report was consistent with another, even earlier study that had observed tumour growth accelerating effects of THC based on reduced tumour immunogenicity [139]. A recent publication further reported that as a result of CB_2_ receptor-induced microglial M2 polarisation, conditioned media of cannabinoid-treated microglial cells increased rather than inhibited the angiogenic capacities of human brain microvascular endothelial cells [140]. The M2 polarisation induced by JWH-133 was demonstrated here by mRNA analyses showing increased levels of the M2 subtype markers CD206, arginase-1 (Arg1) and the chitinase-like protein Ym1 and decreased levels of the M1 subtype markers CD68, CD86 and inducible nitric oxide synthase (iNOS). M2 polarisation also proved to be the mechanism by which the CB_2_ agonist JHW-133 attenuated its own tumour-regressive effect in nude mice with intracranial glioma xenografts, which was only fully expressed by knockdown of cytochrome P450 2J2 (CYP2J2), which mediates the proangiogenic effect [140]. As a functional mediator of CB_2_ receptor-dependent CYP2J2-induced M2 polarisation, the authors pointed to increased synthesis of 11,12-epoxyeicosatrienoic acid (11,12-EET) by CYP2J2.

## Combination partners in anticancer treatments

Although some newly approved anticancer drugs are also used as monotherapy for certain indications, it seems more likely that cannabinoid compounds will be used as a combination and add-on option with currently employed cytostatics, assuming successful clinical trials. Against this background, THC and CBD, which are currently being tested in some studies as combination, have been preclinically shown to enhance the effect of various cytostatics, such as for vinca alkaloids, cytarabine, doxorubicin, mitoxantrone, carmustine, temozolomide, bortezomib, carfilzomib and cisplatin (reviewed in ref. [111, 141]). Thereby, combined administration of CBD and temozolomide in patient-derived neurosphere cultures and orthotopic mouse models was demonstrated to exert a significant synergistic effect in both reducing tumour size and prolonging survival [85]. Of particular importance for the use of cannabinoids in the treatment of glioblastoma is that the aforementioned booster effect on temozolomide action was previously confirmed in elaborate in vivo mouse models [59]. In glioblastoma cells, CBD has also been shown to enhance the effect of cisplatin [142]. Recently, a synergistic effect was also confirmed for the tumour regressive effect of CBD and cisplatin in a murine model of squamous cell carcinoma of the head and neck as well as for the in vitro cytotoxicity of CBD in combination with cisplatin, 5-fluorouracil or paclitaxel on human squamous cell carcinoma cells of the head and neck [52]. However, the mechanisms of these synergies are not yet fully understood. In this context, one study has shown that cannabinoid-mediated enhancement of the effect of vinblastine in resistant leukaemia cells was accompanied by THC- and CBD-induced downregulation of P-glycoprotein [143], while the synergistic cannabinoid effect over mitoxantrone in embryonic fibroblasts occurred via inhibition of ATP-binding cassette transporters (ABC)G2 [144]. Another study focusing on the effect of THC on the sensitisation of leukaemia cells to treatment with cytarabine, doxorubicin and vincristine showed reduced p42/44 MAPK activity as the underlying mechanism of THC-induced enhancement of the respective cytostatic effect [145]. In addition, a number of mechanistic studies have found a CBD-mediated increase in tumour cell susceptibility to the proteasome inhibitor bortezomib [146, 147], doxorubicin [148, 149] as well as temozolomide and carmustine [148]. There has also been a report that CBD increases the uptake into and toxicity on glioma cells of doxorubicin, temozolomide and carmustine via an increase in TRPV2 activity and associated increased calcium influx [148], with these results also confirmed for doxorubicin in triple negative breast cancer cells [149]. With regard to the synergistic effect with bortezomib, it was also shown that the combination of CBD and THC inhibits the expression of the immunoproteasome subunit β5i in multiple myeloma cells [147]. In addition, the synergistic effect of the combination of CBD and THC should also be mentioned here, which for example induces autophagy-dependent necrosis in multiple myeloma cells and inhibits cellular migration by downregulating the expression of the chemokine receptor CXCR4 and the plasma membrane glycoprotein CD147 [147]. However, in contrast to the results of these studies, which showed an enhancement of cytostatic effects when combined with cannabinoid compounds, a recently published paper did not find a survival benefit in the cannabinoid treatment group in combination with cyclophosphamide in an in vivo medulloblastoma model [84].

In addition, several studies suggest that cannabinoid treatment causes glioma cells to become more sensitive to ionising radiation, as shown for the combination of THC and CBD [60] and the combination of CBD with heat shock inhibitors [150]. Increased radiosensitivity was confirmed in CBD-treated glioma cells in another study [151], with the same group later reporting the underlying mechanism to involve inhibition of ATM kinase, a serine/threonine protein kinase that is recruited and activated by DNA double-strand breaks [152].

## Conclusion

Given a considerable number of in vitro and animal studies showing that cannabinoid compounds exert tumour growth inhibitory and antimetastatic effects, cannabinoid compounds may represent a useful additional therapeutic option to currently used cytostatic drugs. This view is also supported by studies indicating a synergistic effect of cannabinoids in combination with currently used chemotherapeutic agents and other therapeutic options. Furthermore, data increasingly suggest that cannabinoids may additionally function as antimetastatic and anti-angiogenic tumour therapy and support the immune system in its defence against tumours.

In addition to the partially divergent preclinical studies already mentioned, some epidemiological studies should also be included in the critical assessment of the potential use of cannabinoids as systemic therapy options. Thus, a recently published prospective observational study showed that cannabis use significantly shortens the time to tumour progression and overall survival of cancer patients [153]. This study illustrates that cannabis use, in this case via modulation of the immune system, can lead to negative and thus life-threatening effects for cancer patients. In addition, a retrospective observational study showed a reduction in the response rate to nivolumab, although the addition of cannabis here had no effect on progression-free survival or overall survival [154]. However, it is worth noting that the administration of cannabinoids in these studies conducted in relatively small patient groups was through the consumption of cannabis oil or smoked/inhaled cannabis, and in many cases the cannabis products were also changed during the course of the study.

On the other hand, these data are counterbalanced by an overwhelming number of studies that clearly show that activation of the EC system is an important factor in tumour defence and thus could serve as a promising target for pharmacological anticancer interventions. In this context, a collection of case reports involving 119 patients also presented impressive examples of breast cancer and glioma patients treated with pharmaceutical-grade synthetic CBD, demonstrating a reduction in circulating tumour cells or a reduction in tumour size by repeat scans [155]. Furthermore, the results of the well-conducted but very small randomised, placebo-controlled phase 1b trial in patients with recurrent glioblastoma multiforme mentioned at the beginning of this article and the higher survival rate shown here in patients taking nabiximols instead of placebo in combination with dose-intense temozolomide [43] provide the rationale for larger and thus adequately powered randomised placebo-controlled trials.

In summary, the property of cannabinoids, in particular, to induce inhibition of tumour growth and spread at multiple levels of tumour progression argues for the use of these substances as an add-on option in tumour treatment. However, it should also be noted that research into the efficacy, dosage and drug safety of cannabinoids in tumour therapy still has a long way to go, especially with regard to clinical trials to be conducted, through which alone the benefits and advantages for cancer patients but also possible risks can be defined.

## Data Availability

Not applicable.

## References

[1] Touwn M (1981). The religious and medicinal uses of Cannabis in China, India and Tibet. J Psychoact Drugs.

[2] Zuardi AW (2006). History of cannabis as a medicine: a review. Braz J Psychiatry.

[3] Liesowska, A Iconic 2,500 year old Siberian princess ‘died from breast cancer’, reveals MRI scan http://siberiantimes.com/science/casestudy/features/iconic-2500-year-old-siberian-princess-died-from-breast-cancer-reveals-unique-mri-scan/ 2014. Accessed on 5 Dec 2021.

[4] Bai Y, Jiang M, Xie T, Jiang C, Gu M, Zhou X (2021). Archaeobotanical evidence of the use of medicinal cannabis in a secular context unearthed from south China. J Ethnopharmacol..

[5] O’Shaughnessy, WB. On the preparations of the Indian hemp or Gunjah, *Transactions of the Medical and Physical Society of Bengal* 1838–1840, p. 421–61. Reprint in: Mikuriya, TH (Ed.): *Marijuana Medical papers* 1839–1972, Medi-Comp Press, Oakland, 1973.

[6] Wood TB, Spivey WTN, Easterfield TH (1899). Cannabinol. Part I. J Chem Soc, Trans.

[7] Mechoulam R, Shvo Y, Hashish I (1963). The structure of cannabidiol. Tetrahedron.

[8] Gaoni Y, Mechoulam R (1964). Isolation, structure, and partial synthesis of an active constituent of hashish. J Am Chem Soc..

[9] Mechoulam R, Gaoni Y (1967). The absolute configuration of delta-1-tetrahydrocannabinol, the major active constituent of hashish. Tetrahedron Lett.

[10] Mechoulam R, Shani A, Edery H, Grunfeld Y (1970). Chemical basis of hashish activity. Science.

[11] Matsuda LA, Lolait SJ, Brownstein MJ, Young AC, Bonner T (1990). Structure of a cannabinoid receptor and functional expression of the cloned cDNA. Nature.

[12] Munro S, Thomas KL, Abu-Shaar M (1993). Molecular characterization of a peripheral receptor for cannabinoids. Nature.

[13] Devane WA, Hanus L, Breuer A, Pertwee RG, Stevenson LA, Griffin G (1992). Isolation and structure of a brain constituent that binds to the cannabinoid receptor. Science.

[14] Mechoulam R, Ben-Shabat S, Hanus L, Ligumsky M, Kaminski NE, Schatz AR (1995). Identification of an endogenous 2-monoglyceride, present in canine gut, that binds to cannabinoid receptors. Biochem Pharmacol.

[15] Schwarz R, Ramer R, Hinz B (2018). Targeting the endocannabinoid system as a potential anticancer approach. Drug Metab Rev..

[16] Zygmunt PM, Petersson J, Andersson DA, Chuang H, Sørgård M, Di Marzo V (1999). Vanilloid receptors on sensory nerves mediate the vasodilator action of anandamide. Nature.

[17] Bisogno T, Hanus L, De Petrocellis L, Tchilibon S, Ponde DE, Brandi I (2001). Molecular targets for cannabidiol and its synthetic analogues: effect on vanilloid VR1 receptors and on the cellular uptake and enzymatic hydrolysis of anandamide. Br J Pharmacol.

[18] Pertwee RG (2008). The diverse CB_1_ and CB_2_ receptor pharmacology of three plant cannabinoids: Δ^9^-tetrahydrocannabinol, cannabidiol and Δ^9^-tetrahydrocannabivarin. Br J Pharmacol.

[19] Ryberg E, Larsson N, Sjögren S, Hjorth S, Hermansson NO, Leonova J (2007). The orphan receptor GPR55 is a novel cannabinoid receptor. Br J Pharmacol..

[20] Rosenthaler S, Pöhn B, Kolmanz C, Huu CN, Krewenka C, Huber A (2014). Differences in receptor binding affinity of several phytocannabinoids do not explain their effects on neural cell cultures. Neurotoxicol Teratol.

[21] O’Sullivan SE, Sun Y, Bennett AJ, Randall MD, Kendall DA (2009). Time-dependent vascular actions of cannabidiol in the rat aorta. Eur J Pharmacol..

[22] Di Marzo V (2009). The endocannabinoid system: its general strategy of action, tools for its pharmacological manipulation and potential therapeutic exploitation. Pharmacol Res..

[23] Deutsch DG, Chin SA (1993). Enzymatic synthesis and degradation of anandamide, a cannabinoid receptor agonist. Biochem Pharmacol..

[24] Blankman JL, Simon GM, Cravatt BF (2007). A comprehensive profile of brain enzymes that hydrolyze the endocannabinoid 2-arachidonoylglycerol. Chem Biol..

[25] Di Marzo V (2018). New approaches and challenges to targeting the endocannabinoid system. Nat Rev Drug Discov..

[26] Munson AE, Harris LS, Friedman MA, Dewey WL, Carchman RA (1975). Antineoplastic activity of cannabinoids. J Natl Cancer Inst..

[27] Galve-Roperh I, Sánchez C, Cortés ML, Gómez del Pulgar T, Izquierdo M, Guzmán M (2000). Anti-tumoural action of cannabinoids, involvement of sustained ceramide accumulation and extracellular signal-regulated kinase activation. Nat Med.

[28] Sánchez C, de Ceballos ML, Gomez del Pulgar T, Rueda D, Corbacho C, Velasco G (2001). Inhibition of glioma growth in vivo by selective activation of the CB_2_ cannabinoid receptor. Cancer Res..

[29] Long JZ, Li W, Booker L, Burston JJ, Kinsey SG, Schlosburg JE (2009). Selective blockade of 2-arachidonoylglycerol hydrolysis produces cannabinoid behavioral effects. Nat Chem Biol.

[30] Nomura DK, Long JZ, Niessen S, Hoover HS, Ng S-W, Cravatt BF (2010). Monoacylglycerol lipase regulates a fatty acid network that promotes cancer pathogenesis. Cell.

[31] Nomura DK, Lombardi DP, Chang JW, Niessen S, Ward AM, Long JZ (2011). Monoacylglycerol lipase exerts dual control over endocannabinoid and fatty acid pathways to support prostate cancer. Chem Biol.

[32] Prüser JL, Ramer R, Wittig F, Ivanov I, Merkord J, Hinz B (2021). The monoacylglycerol lipase inhibitor JZL184 inhibits lung cancer cell invasion and metastasis via the CB_1_ cannabinoid receptor. Mol Cancer Ther..

[33] Liu R, Wang X, Curtiss C, Landas S, Rong R, Sheikh MS (2018). Monoglyceride lipase gene knockout in mice leads to increased incidence of lung adenocarcinoma. Cell Death Dis.

[34] Ligresti A, Bisogno T, Matias I, De Petrocellis L, Cascio MG, Cosenza V (2003). Possible endocannabinoid control of colorectal cancer growth. Gastroenterology.

[35] Bifulco M, Laezza C, Valenti M, Ligresti A, Portella G, Di Marzo V (2004). A new strategy to block tumour growth by inhibiting endocannabinoid inactivation. FASEB J.

[36] Winkler K, Ramer R, Dithmer S, Ivanov I, Merkord J, Hinz B (2016). Fatty acid amide hydrolase inhibitors confer anti-invasive and antimetastatic effects on lung cancer cells. Oncotarget.

[37] Gertsch J, Leonti M, Raduner S, Racz I, Chen JZ, Xie XQ (2008). Beta-caryophyllene is a dietary cannabinoid. Proc Natl Acad Sci USA.

[38] Dahham SS, Tabana YM, Iqbal MA, Ahamed MB, Ezzat MO, Majid AS (2015). The anticancer, antioxidant and antimicrobial properties of the sesquiterpene β-caryophyllene from the essential oil of aquilaria crassna. Molecules.

[39] Irrera N, D’Ascola A, Pallio G, Bitto A, Mannino F, Arcoraci V (2020). β-Caryophyllene inhibits cell proliferation through a direct modulation of CB_2_ receptors in glioblastoma cells. Cancers (Basel).

[40] Di Giacomo S, Di Sotto A, Mazzanti G, Wink M (2017). Chemosensitizing properties of β-caryophyllene and β-caryophyllene oxide in combination with doxorubicin in human cancer cells. Anticancer Res..

[41] Di Giacomo S, Briz O, Monte MJ, Sanchez-Vicente L, Abete L, Lozano E (2019). Chemosensitization of hepatocellular carcinoma cells to sorafenib by β-caryophyllene oxide-induced inhibition of ABC export pumps. Arch Toxicol..

[42] Guzmán M, Duarte MJ, Blázquez C, Ravina J, Rosa MC, Galve-Roperh I (2006). A pilot clinical study of Δ^9^-tetrahydrocannabinol in patients with recurrent glioblastoma multiforme. Br J Cancer.

[43] Twelves C, Sabel M, Checketts D, Miller S, Tayo B, Jove M (2021). GWCA1208 study group. A phase 1b randomised, placebo-controlled trial of nabiximols cannabinoid oromucosal spray with temozolomide in patients with recurrent glioblastoma. Br J Cancer.

[44] Blázquez C, Carracedo A, Barrado L, Real PJ, Fernández-Luna JL, Velasco G (2006). Cannabinoid receptors as novel targets for the treatment of melanoma. FASEB J..

[45] Caffarel MM, Andradas C, Mira E, Pérez-Gómez E, Cerutti C, Moreno-Bueno G (2010). Cannabinoids reduce ErbB2-driven breast cancer progression through Akt inhibition. Mol Cancer.

[46] Xian XS, Park H, Cho YK, Lee IS, Kim SW, Choi MG (2010). Effect of a synthetic cannabinoid agonist on the proliferation and invasion of gastric cancer cells. J Cell Biochem..

[47] Boyacıoğlu Ö, Bilgiç E, Varan C, Bilensoy E, Nemutlu E, Sevim D (2021). ACPA decreases non-small cell lung cancer line growth through Akt/PI3K and JNK pathways in vitro. Cell Death Dis..

[48] Rao M, Chen D, Zhan P, Jiang J (2019). MDA19 a novel CB_2_ agonist inhibits hepatocellular carcinoma partly through inactivation of AKT signaling pathway. Biol Direct..

[49] Caffarel MM, Sarrió D, Palacios J, Guzmán M, Sánchez C (2006). Δ^9^-Tetrahydrocannabinol inhibits cell cycle progression in human breast cancer cells through Cdc2 regulation. Cancer Res..

[50] Laezza C, Pisanti S, Crescenzi E, Bifulco M (2006). Anandamide inhibits Cdk2 and activates Chk1 leading to cell cycle arrest in human breast cancer cells. FEBS Lett.

[51] Roberto D, Klotz LH, Venkateswaran V (2019). Cannabinoid WIN 55,212-2 induces cell cycle arrest and apoptosis, and inhibits proliferation, migration, invasion, and tumor growth in prostate cancer in a cannabinoid-receptor 2 dependent manner. Prostate.

[52] Go YY, Kim SR, Kim DY, Chae SW, Song JJ (2020). Cannabidiol enhances cytotoxicity of anti-cancer drugs in human head and neck squamous cell carcinoma. Sci Rep..

[53] Zhang X, Qin Y, Pan Z, Li M, Liu X, Chen X (2019). Cannabidiol induces cell cycle arrest and cell apoptosis in human gastric cancer SGC-7901 cells. Biomolecules.

[54] Massi P, Valenti M, Vaccani A, Gasperi V, Perletti G, Marras E (2008). 5-Lipoxygenase and anandamide hydrolase (FAAH) mediate the antitumor activity of cannabidiol, a non-psychoactive cannabinoid. J Neurochem..

[55] Liu C, Sadat SH, Ebisumoto K, Sakai A, Panuganti BA, Ren S (2020). Cannabinoids promote progression of HPV-positive head and neck squamous cell carcinoma via p38 MAPK activation. Clin Cancer Res..

[56] Hart S, Fischer OM, Ullrich A (2004). Cannabinoids induce cancer cell proliferation via tumor necrosis factor alpha-converting enzyme (TACE/ADAM17)-mediated transactivation of the epidermal growth factor receptor. Cancer Res..

[57] Miyato H, Kitayama J, Yamashita H, Souma D, Asakage M, Yamada J (2009). Pharmacological synergism between cannabinoids and paclitaxel in gastric cancer cell lines. J Surg Res..

[58] Martínez-Martínez E, Martín-Ruiz A, Martín P, Calvo V, Provencio M, García JM (2016). CB_2_ cannabinoid receptor activation promotes colon cancer progression via AKT/GSK3β signaling pathway. Oncotarget.

[59] Torres S, Lorente M, Rodríguez-Fornés F, Hernández-Tiedra S, Salazar M, García-Taboada E (2011). A combined preclinical therapy of cannabinoids and temozolomide against glioma. Mol Cancer Ther..

[60] Scott KA, Dalgleish AG, Liu WM (2014). The combination of cannabidiol and ∆^9^-tetrahydrocannabinol enhances the anticancer effects of radiation in an orthotopic murine glioma model. Mol Cancer Ther..

[61] Fowler CJ (2015). Delta^9^-tetrahydrocannabinol and cannabidiol as potential curative agents for cancer: a critical examination of the preclinical literature. Clin Pharmacol Ther..

[62] Gómez del Pulgar T, Velasco G, Sánchez C, Haro A, Guzmán M (2002). De novo-synthesized ceramide is involved in cannabinoid-induced apoptosis. Biochem J..

[63] Salazar M, Carracedo A, Salanueva IJ, Hernández-Tiedra S, Lorente M, Egia A (2009). Cannabinoid action induces autophagy-mediated cell death through stimulation of ER stress in human glioma cells. J Clin Invest..

[64] Carracedo A, Gironella M, Lorente M, Garcia S, Guzmán M, Velasco G (2006). Cannabinoids induce apoptosis of pancreatic tumour cells via endoplasmic reticulum stress-related genes. Cancer Res.

[65] Oesch S, Walter D, Wachtel M, Pretre K, Salazar M, Guzmán M (2009). Cannabinoid receptor 1 is a potential drug target for treatment of translocation-positive rhabdomyosarcoma. Mol Cancer Ther..

[66] Ramer R, Weinzierl U, Schwind B, Brune K, Hinz B (2003). Ceramide is involved in *R*(+)-methanandamide-induced cyclooxygenase-2 expression in human neuroglioma cells. Mol Pharmacol..

[67] Hinz B, Ramer R, Eichele K, Weinzierl U, Brune K (2004). Up-regulation of cyclooxygenase-2 expression is involved in *R*(+)-methanandamide-induced apoptotic death of human neuroglioma cells. Mol Pharmacol.

[68] Eichele K, Ramer R, Hinz B (2009). R(+)-methanandamide-induced apoptosis of human cervical carcinoma cells involves a cyclooxygenase-2-dependent pathway. Pharm Res..

[69] Eichele K, Ramer R, Hinz B (2008). Decisive role of cyclooxygenase-2 and lipocalin-type prostaglandin D synthase in chemotherapeutics-induced apoptosis of human cervical carcinoma cells. Oncogene.

[70] Ramer R, Heinemann K, Merkord J, Rohde H, Salamon A, Linnebacher M (2013). COX-2 and PPAR-γ confer cannabidiol-induced apoptosis of human lung cancer cells. Mol Cancer Ther.

[71] Maccarrone M, Lorenzon T, Bari M, Melino G, Finazzi-Agro A (2000). Anandamide induces apoptosis in human cells via vanilloid receptors. Evidence for a protective role of cannabinoid receptors. J Biol Chem..

[72] Soliman E, Van Dross R (2016). Anandamide-induced endoplasmic reticulum stress and apoptosis are mediated by oxidative stress in non-melanoma skin cancer: Receptor-independent endocannabinoid signaling. Mol Carcinog..

[73] Huang L, Ramirez JC, Frampton GA, Golden LE, Quinn MA, Pae HY (2011). Anandamide exerts its antiproliferative actions on cholangiocarcinoma by activation of the GPR55 receptor. Lab Invest..

[74] Yamada T, Ueda T, Shibata Y, Ikegami Y, Saito M, Ishida Y (2010). TRPV2 activation induces apoptotic cell death in human T24 bladder cancer cells: a potential therapeutic target for bladder cancer. Urology.

[75] Shrivastava A, Kuzontkoski PM, Groopman JE, Prasad A (2011). Cannabidiol induces programmed cell death in breast cancer cells by coordinating the cross-talk between apoptosis and autophagy. Mol Cancer Ther..

[76] McKallip RJ, Jia W, Schlomer J, Warren JW, Nagarkatti PS, Nagarkatti M (2006). Cannabidiol-induced apoptosis in human leukemia cells: A novel role of cannabidiol in the regulation of p22phox and Nox4 expression. Mol Pharmacol.

[77] Jeong S, Jo MJ, Yun HK, Kim DY, Kim BR, Kim JL (2019). Cannabidiol promotes apoptosis via regulation of XIAP/Smac in gastric cancer. Cell Death Dis..

[78] Olivas-Aguirre M, Torres-López L, Valle-Reyes JS, Hernández-Cruz A, Pottosin I, Dobrovinskaya O (2019). Cannabidiol directly targets mitochondria and disturbs calcium homeostasis in acute lymphoblastic leukemia. Cell Death Dis..

[79] Vara D, Salazar M, Olea-Herrero N, Guzmán M, Velasco G, Díaz-Laviada I (2011). Anti-tumoural action of cannabinoids on hepatocellular carcinoma, role of AMPK-dependent activation of autophagy. Cell Death Differ.

[80] Armstrong JL, Hill DS, McKee CS, Hernandez-Tiedra S, Lorente M, Lopez-Valero I (2015). Exploiting cannabinoid-induced cytotoxic autophagy to drive melanoma cell death. J Invest Dermatol.

[81] Ellert-Miklaszewska A, Ciechomska IA, Kaminska B (2021). Synthetic cannabinoids induce autophagy and mitochondrial apoptotic pathways in human glioblastoma cells independently of deficiency in *TP53* or *PTEN* tumour suppressors. Cancers (Basel).

[82] Ivanov VN, Grabham PW, Wu CC, Hei TK (2020). Inhibition of autophagic flux differently modulates cannabidiol-induced death in 2D and 3D glioblastoma cell cultures. Sci Rep..

[83] Nabissi M, Morelli MB, Amantini C, Liberati S, Santoni M, Ricci-Vitiani L (2015). Cannabidiol stimulates Aml-1a-dependent glial differentiation and inhibits glioma stem-like cells proliferation by inducing autophagy in a TRPV2-dependent manner. Int J Cancer.

[84] Andradas C, Byrne J, Kuchibhotla M, Ancliffe M, Jones AC, Carline B (2021). Assessment of cannabidiol and Δ^9^-tetrahydrocannabiol in mouse models of medulloblastoma and ependymoma. Cancers (Basel).

[85] Huang T, Xu T, Wang Y, Zhou Y, Yu D, Wang Z (2021). Cannabidiol inhibits human glioma by induction of lethal mitophagy through activating TRPV4. Autophagy.

[86] Meyer N, Zielke S, Michaelis JB, Linder B, Warnsmann V, Rakel S (2018). AT 101 induces early mitochondrial dysfunction and HMOX1 (heme oxygenase 1) to trigger mitophagic cell death in glioma cells. Autophagy.

[87] Nithipatikom K, Endsley MP, Isbell MA, Falck JR, Iwamoto Y, Hillard CJ (2004). 2-arachidonoylglycerol, a novel inhibitor of androgen-independent prostate cancer cell invasion. Cancer Res..

[88] Ma C, Wu TT, Jiang PC, Li ZQ, Chen XJ, Fu K (2016). Anti-carcinogenic activity of anandamide on human glioma in vitro and in vivo. Mol Med Rep..

[89] Ramer R, Hinz B (2008). Inhibition of cancer cell invasion by cannabinoids via increased expression of tissue inhibitor of matrix metalloproteinases-1. J Natl Cancer Inst..

[90] Ramer R, Merkord J, Rohde H, Hinz B (2010). Cannabidiol inhibits cancer cell invasion via upregulation of tissue inhibitor of matrix metalloproteinases-1. Biochem Pharmacol.

[91] Stamenkovic I (2000). Matrix metalloproteinases in tumor invasion and metastasis. Semin Cancer Biol.

[92] Cruz-Munoz W, Khokha R (2008). The role of tissue inhibitors of metalloproteinases in tumorigenesis and metastasis. Crit Rev Clin Lab Sci..

[93] Ramer R, Bublitz K, Freimuth N, Merkord J, Rohde H, Haustein M (2012). Cannabidiol inhibits lung cancer cell invasion and metastasis via intercellular adhesion molecule-1. FASEB J..

[94] Blázquez C, Salazar M, Carracedo A, Lorente M, Egia A, González-Feria L (2008). Cannabinoids inhibit glioma cell invasion by down-regulating matrix metalloproteinase-2 expression. Cancer Res..

[95] Pourkhalili N, Ghahremani MH, Farsandaj N, Tavajohi S, Majdzadeh M, Parsa M (2013). Evaluation of anti-invasion effect of cannabinoids on human hepatocarcinoma cells. Toxicol Mech Methods.

[96] Notaro A, Emanuele S, Geraci F, D’Anneo A, Lauricella M, Calvaruso G (2019). WIN55212-2-induced expression of mir-29b1 favours the suppression of osteosarcoma cell migration in a SPARC-independent manner. Int J Mol Sci.

[97] Pietrovito L, Iozzo M, Bacci M, Giannoni E, Chiarugi P (2020). Treatment with cannabinoids as a promising approach for impairing fibroblast activation and prostate cancer progression. Int J Mol Sci..

[98] McAllister SD, Christian RT, Horowitz MP, Garcia A, Desprez PY (2007). Cannabidiol as a novel inhibitor of Id-1 gene expression in aggressive breast cancer cells. Mol Cancer Ther..

[99] Soroceanu L, Murase R, Limbad C, Singer E, Allison J, Adrados I (2013). Id-1 is a key transcriptional regulator of glioblastoma aggressiveness and a novel therapeutic target. Cancer Res..

[100] Murase R, Sumida T, Kawamura R, Onishi-Ishikawa A, Hamakawa H, McAllister SD (2016). Suppression of invasion and metastasis in aggressive salivary cancer cells through targeted inhibition of ID1 gene expression. Cancer Lett..

[101] McAllister SD, Murase R, Christian RT, Lau D, Zielinski AJ, Allison J (2011). Pathways mediating the effects of cannabidiol on the reduction of breast cancer cell proliferation invasion and metastasis. Breast Cancer Res Treat..

[102] Murase R, Kawamura R, Singer E, Pakdel A, Sarma P, Judkins J (2014). Targeting multiple cannabinoid anti-tumour pathways with a resorcinol derivative leads to inhibition of advanced stages of breast cancer. Br J Pharmacol.

[103] Ramer R, Rohde A, Merkord J, Rohde H, Hinz B (2010). Decrease of plasminogen activator inhibitor-1 may contribute to the anti-invasive action of cannabidiol on human lung cancer cells. Pharm Res.

[104] Leelawat S, Leelawat K, Narong S, Matangkasombut O (2010). The dual effects of Δ^9^-tetrahydrocannabinol on cholangiocarcinoma cells, anti-invasion activity at low concentration and apoptosis induction at high concentration. Cancer Invest..

[105] Anis O, Vinayaka AC, Shalev N, Namdar D, Nadarajan S, Anil SM (2021). Cannabis-derived compounds cannabichromene and Δ^9^-tetrahydrocannabinol interact and exhibit cytotoxic activity against urothelial cell carcinoma correlated with inhibition of cell migration and cytoskeleton organization. Molecules.

[106] Xu S, Ma H, Bo Y, Shao M (2019). The oncogenic role of CB_2_ in the progression of non-small-cell lung cancer. Biomed Pharmacother..

[107] Coke CJ, Scarlett KA, Chetram MA, Jones KJ, Sandifer BJ, Davis AS (2016). Simultaneous activation of induced heterodimerization between CXCR4 chemokine receptor and cannabinoid receptor 2 (CB_2_) reveals a mechanism for regulation of tumour progression. J Biol Chem..

[108] Laezza C, D’Alessandro A, Paladino S, Malfitano AM, Proto MC, Gazzerro P (2012). Anandamide inhibits the Wnt/β-catenin signalling pathway in human breast cancer MDA MB 231 cells. Eur J Cancer.

[109] García-Morales L, Castillo AM, Tapia Ramírez J, Zamudio-Meza H, Domínguez-Robles MDC, Meza I (2020). CBD reverts the mesenchymal invasive phenotype of breast cancer cells induced by the inflammatory cytokine IL-1β. Int J Mol Sci..

[110] Milian L, Mata M, Alcacer J, Oliver M, Sancho-Tello M, Martín de Llano JJ (2020). Cannabinoid receptor expression in non-small cell lung cancer. Effectiveness of tetrahydrocannabinol and cannabidiol inhibiting cell proliferation and epithelial-mesenchymal transition in vitro. PLoS ONE.

[111] Ramer R, Hinz B (2017). Cannabinoids as anticancer drugs. Adv Pharmacol..

[112] Portella G, Laezza C, Laccetti P, De Petrocellis L, Di Marzo V, Bifulco M (2003). Inhibitory effects of cannabinoid CB_1_ receptor stimulation on tumor growth and metastatic spreading: actions on signals involved in angiogenesis and metastasis. FASEB J..

[113] Preet A, Ganju RK, Groopman JE (2008). Δ^9^-Tetrahydrocannabinol inhibits epithelial growth factor-induced lung cancer cell migration in vitro as well as its growth and metastasis in vivo. Oncogene.

[114] Qamri Z, Preet A, Nasser MW, Bass CE, Leone G, Barsky SH (2009). Synthetic cannabinoid receptor agonists inhibit tumor growth and metastasis of breast cancer. Mol Cancer Ther..

[115] Marino S, de Ridder D, Bishop RT, Renema N, Ponzetti M, Sophocleous A (2019). Paradoxical effects of JZL184, an inhibitor of monoacylglycerol lipase, on bone remodelling in healthy and cancer-bearing mice. EBioMedicine.

[116] Hu WR, Lian YF, Peng LX, Lei JJ, Deng CC, Xu M (2014). Monoacylglycerol lipase promotes metastases in nasopharyngeal carcinoma. Int J Clin Exp Pathol.

[117] Ramer R, Hinz B (2015). New insights into antimetastatic and antiangiogenic effects of cannabinoids. Int Rev Cell Mol Biol..

[118] Casanova ML, Blázquez C, Martínez-Palacio J, Villanueva C, Fernández-Aceñero MJ, Huffman JW (2003). Inhibition of skin tumour growth and angiogenesis in vivo by activation of cannabinoid receptors. J Clin Invest.

[119] Blázquez C, Casanova ML, Planas A, Gómez Del Pulgar T, Villanueva C, Fernández-Aceñero MJ (2003). Inhibition of tumour angiogenesis by cannabinoids. FASEB J..

[120] Blázquez C, González-Feria L, Alvarez L, Haro A, Casanova ML, Guzmán M (2004). Cannabinoids inhibit the vascular endothelial growth factor pathway in gliomas. Cancer Res..

[121] Solinas M, Massi P, Cantelmo AR, Cattaneo MG, Cammarota R, Bartolini D (2012). Cannabidiol inhibits angiogenesis by multiple mechanisms. Br J Pharmacol..

[122] Thapa D, Lee JS, Heo SW, Lee YR, Kang KW, Kwak MK (2011). Novel hexahydrocannabinol analogs as potential anti-cancer agents inhibit cell proliferation and tumour angiogenesis. Eur J Pharmacol.

[123] Picardi P, Ciaglia E, Proto M, Pisanti S (2014). Anandamide inhibits breast tumour-induced angiogenesis. Transl Med UniSa.

[124] Ramer R, Fischer S, Haustein M, Manda K, Hinz B (2014). Cannabinoids inhibit angiogenic capacities of endothelial cells via release of tissue inhibitor of matrix metalloproteinases-1 from lung cancer cells. Biochem Pharmacol.

[125] Braile M, Cristinziano L, Marcella S, Varricchi G, Marone G, Modestino L (2021). LPS-mediated neutrophil VEGF-A release is modulated by cannabinoid receptor activation. J Leukoc Biol.

[126] Pisanti S, Picardi P, Prota L, Proto MC, Laezza C, McGuire PG (2011). Genetic and pharmacologic inactivation of cannabinoid CB_1_ receptor inhibits angiogenesis. Blood.

[127] Pisanti S, Borselli C, Oliviero O, Laezza C, Gazzerro P, Bifulco M (2007). Antiangiogenic activity of the endocannabinoid anandamide, correlation to its tumour-suppressor efficacy. J Cell Physiol..

[128] Kogan NM, Blázquez C, Alvarez L, Gallily R, Schlesinger M, Guzmán M (2006). A cannabinoid quinone inhibits angiogenesis by targeting vascular endothelial cells. Mol Pharmacol..

[129] Hofmann NA, Barth S, Waldeck-Weiermair M, Klec C, Strunk D, Malli R (2014). TRPV1 mediates cellular uptake of anandamide and thus promotes endothelial cell proliferation and network-formation. Biol Open.

[130] Böckmann S, Hinz B (2020). Cannabidiol promotes endothelial cell survival by heme oxygenase-1-mediated autophagy. Cells.

[131] Aird WC (2012). Endothelial cell heterogeneity. Cold Spring Harb Perspect Med.

[132] Hu Y, Ranganathan M, Shu C, Liang X, Ganesh S, Osafo-Addo A (2020). Single-cell transcriptome mapping identifies common and cell-type specific genes affected by acute delta9-tetrahydrocannabinol in humans. Sci Rep..

[133] Yang Y, Huynh N, Dumesny C, Wang K, He H, Nikfarjam M (2020). Cannabinoids inhibited pancreatic cancer via P-21 activated kinase 1 mediated pathway. Int J Mol Sci.

[134] Qiu C, Yang L, Wang B, Cui L, Li C, Zhuo Y (2019). The role of 2-arachidonoylglycerol in the regulation of the tumour-immune microenvironment in murine models of pancreatic cancer. Biomed Pharmacother..

[135] Glodde N, Jakobs M, Bald T, Tüting T, Gaffal E (2015). Differential role of cannabinoids in the pathogenesis of skin cancer. Life Sci.

[136] Haustein M, Ramer R, Linnebacher M, Manda K, Hinz B (2014). Cannabinoids increase lung cancer cell lysis by lymphokine-activated killer cells via upregulation of ICAM-1. Biochem Pharmacol..

[137] Sekiba K, Otsuka M, Seimiya T, Tanaka E, Funato K, Miyakawa Y (2020). The fatty-acid amide hydrolase inhibitor URB597 inhibits MICA/B shedding. Sci Rep..

[138] McKallip RJ, Nagarkatti M, Nagarkatti PS (2005). Δ-9-tetrahydrocannabinol enhances breast cancer growth and metastasis by suppression of the antitumour immune response. J Immunol.

[139] Zhu LX, Sharma S, Stolina M, Gardner B, Roth MD, Tashkin DP (2000). Δ-9-tetrahydrocannabinol inhibits antitumour immunity by a CB_2_ receptor-mediated, cytokine-dependent pathway. J Immunol.

[140] Lei X, Chen X, Quan Y, Tao Y, Li J (2020). Targeting CYP2J2 to enhance the anti-glioma efficacy of cannabinoid receptor 2 stimulation by inhibiting the pro-angiogenesis function of M2 microglia. Front Oncol..

[141] Hinz B, Ramer R (2019). Anti-tumour actions of cannabinoids. Br J Pharmacol..

[142] Deng L, Ng L, Ozawa T, Stella N (2017). Quantitative analyses of synergistic responses between cannabidiol and DNA-damaging agents on the proliferation and viability of glioblastoma and neural progenitor cells in culture. J Pharmacol Exp Ther.

[143] Holland ML, Panetta JA, Hoskins JM, Bebawy M, Roufogalis BD, Allen JD (2006). The effects of cannabinoids on P-glycoprotein transport and expression in multidrug resistant cells. Biochem Pharmacol.

[144] Holland ML, Lau DT, Allen JD, Arnold JC (2007). The multidrug transporter ABCG2 (BCRP) is inhibited by plant-derived cannabinoids. Br J Pharmacol..

[145] Liu WM, Scott KA, Shamash J, Joel S, Powles TB (2008). Enhancing the in vitro cytotoxic activity of Δ^9^-tetrahydrocannabinol in leukemic cells through a combinatorial approach. Leuk Lymphoma.

[146] Morelli MB, Offidani M, Alesiani F, Discepoli G, Liberati S, Olivieri A (2014). The effects of cannabidiol and its synergism with bortezomib in multiple myeloma cell lines. A role for transient receptor potential vanilloid type-2. Int J Cancer.

[147] Nabissi M, Morelli MB, Offidani M, Amantini C, Gentili S, Soriani A (2016). Cannabinoids synergize with carfilzomib, reducing multiple myeloma cells viability and migration. Oncotarget.

[148] Nabissi M, Morelli MB, Santoni M, Santoni G (2013). Triggering of the TRPV2 channel by cannabidiol sensitizes glioblastoma cells to cytotoxic chemotherapeutic agents. Carcinogenesis.

[149] Elbaz M, Ahirwar D, Xiaoli Z, Zhou X, Lustberg M, Nasser MW (2016). TRPV2 is a novel biomarker and therapeutic target in triple negative breast cancer. Oncotarget.

[150] Scott KA, Dennis JL, Dalgleish AG, Liu WM (2015). Inhibiting heat shock proteins can potentiate the cytotoxic effect of cannabidiol in human glioma cells. Anticancer Res.

[151] Ivanov VN, Wu J, Hei TK (2017). Regulation of human glioblastoma cell death by combined treatment of cannabidiol, γ-radiation and small molecule inhibitors of cell signaling pathways. Oncotarget.

[152] Ivanov VN, Wu J, Wang TJC, Hei TK (2019). Inhibition of ATM kinase upregulates levels of cell death induced by cannabidiol and γ-irradiation in human glioblastoma cells. Oncotarget.

[153] Bar-Sela G, Cohen I, Campisi-Pinto S, Lewitus GM, Oz-Ari L, Jehassi A (2020). Cannabis consumption used by cancer patients during immunotherapy correlates with poor clinical outcome. Cancers (Basel).

[154] Taha T, Meiri D, Talhamy S, Wollner M, Peer A, Bar-Sela G (2019). Cannabis impacts tumour response rate to nivolumab in patients with advanced malignancies. Oncologist.

[155] Kenyon J, Liu W, Dalgleish A (2018). Report of objective clinical responses of cancer patients to pharmaceutical-grade synthetic cannabidiol. Anticancer Res..

